# Behavior of platinum(iv) complexes in models of tumor hypoxia: cytotoxicity, compound distribution and accumulation†

**DOI:** 10.1039/c5mt00312a

**Published:** 2016-02-10

**Authors:** Ekaterina Schreiber-Brynzak, Verena Pichler, Petra Heffeter, Buck Hanson, Sarah Theiner, Irene Lichtscheidl-Schultz, Christoph Kornauth, Luca Bamonti, Vineet Dhery, Diana Groza, David Berry, Walter Berger, Markus Galanski, Michael A. Jakupec, Bernhard K. Keppler

**Affiliations:** a Institute of Inorganic Chemistry, University of Vienna, Waehringer Strasse 42, 1090 Vienna, Austria; b Department of Medicine I, Institute of Cancer Research and Comprehensive Cancer Center, Medical University of Vienna, Borschkegasse 8a, 1090 Vienna, Austria; c Research Platform “Translational Cancer Therapy Research”, University of Vienna, Waehringer Strasse 42, 1090 Vienna, Austria; d Department of Microbiology and Ecosystem Science, Division of Microbial Ecology, University of Vienna, Althanstrasse 14 (UZA I), 1090 Vienna, Austria; e Research Network Chemistry Meets Microbiology, University of Vienna, Althanstrasse 14 (UZA I), 1090 Vienna, Austria; f Core Facility Cell Imaging and Ultrastructure Research, University of Vienna, Althanstrasse 14 (UZA I), 1090 Vienna, Austria; g Clinical Institute of Pathology, Vienna General Hospital, Medical University of Vienna, Spitalgasse 23, 1090 Vienna, Austria

## Abstract

Hypoxia in solid tumors remains a challenge for conventional cancer therapeutics. As a source for resistance, metastasis development and drug bioprocessing, it influences treatment results and disease outcome. Bioreductive platinum(iv) prodrugs might be advantageous over conventional metal-based therapeutics, as biotransformation in a reductive milieu, such as under hypoxia, is required for drug activation. This study deals with a two-step screening of experimental platinum(iv) prodrugs with different rates of reduction and lipophilicity with the aim of identifying the most appropriate compounds for further investigations. In the first step, the cytotoxicity of all compounds was compared in hypoxic multicellular spheroids and monolayer culture using a set of cancer cell lines with different sensitivities to platinum(ii) compounds. Secondly, two selected compounds were tested in hypoxic xenografts in SCID mouse models in comparison to satraplatin, and, additionally, (LA)-ICP-MS-based accumulation and distribution studies were performed for these compounds in hypoxic spheroids and xenografts. Our findings suggest that, while cellular uptake and cytotoxicity strongly correlate with lipophilicity, cytotoxicity under hypoxia compared to non-hypoxic conditions and antitumor activity of platinum(iv) prodrugs are dependent on their rate of reduction.

## Introduction

Since its approval for cancer therapy in the late 1970s, cisplatin has been used so widely that it is often called “penicillin of cancer”. The continued success of this chemotherapeutic drug inspired the further development of platinum(ii) compounds for anticancer therapy, generating hundreds of cisplatin analogs. Beside cisplatin, oxaliplatin and carboplatin are the only metal-based anticancer drugs widely established in clinical use, while others, such as picoplatin, are currently undergoing clinical trials. However, major limitations of platinum therapy, such as resistance development and dose-limiting side effects, remain.^1^–^4^ Some attempts to overcome the disadvantages of cisplatin treatment were made by exploring platinum(iv) compounds. The most prominent representative of platinum(iv)-based prodrugs, satraplatin, was undergoing different phase I, II, and III clinical trials as single and combination therapy for different cancer types, but most of the trials revealed moderate to low activity compared to standard treatment.^5^–^7^ Nevertheless, platinum(iv) complexes are promising anticancer agents, as they show high stability and bioavailability, allowing oral application and overcoming side effects such as nephrotoxicity.^8^,^9^ Platinum(iv) complexes have to be activated by reduction to release their active platinum(ii) metabolites. Therefore, platinum(iv) complexes, as bioreductive prodrugs, might target hypoxia and close the gap between classic metal-based and targeted therapies.^10^–^12^

Hypoxia, an important characteristic of progressive solid tumors, has been a challenge for conventional anticancer therapy for decades.^13^–^17^ As most chemotherapeutics, including platinum(ii) drugs, target highly proliferating cancer cells, the strong quiescent cell fraction typically associated with hypoxia is hardly affected by many treatments.^18^,^19^ Additionally, necrotic and hypoxic regions generate inhomogeneities in tumor tissue, with changing physico-chemical and biological properties, such as pH and pO_2_ gradients and different protein expression patterns.^20^ This bioreductive environment influences the stability of chemotherapeutic agents and can cause specific biotransformation of these drugs. In this case, platinum(iv) complexes might have a substantial advantage over other therapeutic approaches, since they first have to be reduced for drug activation. Nevertheless, the activation of platinum(iv) species in hypoxic tumors has barely been investigated so far.

Hall *et al.* and Mellor *et al.* suggested that platinum(iv) complexes may be reduced to their active platinum(ii) counterparts under conditions of solid tumors.^21^,^23^ Different small-molecular weight reducing agents, such as ascorbic acid, glutathione, or amino acids such as l-methionine and l-cysteine, but also proteins such as metallothioneins and albumin were discussed to be responsible for intra- as well as extracellular reduction *in vivo* and *in vitro*.^22^ However, the exact mechanism of platinum(iv) reduction remains unknown and attempts to investigate the influence of the tumor micro-environment on platinum(iv) complex stability were not fully conclusive.^23^ Nevertheless, the hypothesis of a possible reduction of platinum(iv) compounds under hypoxic conditions in solid tumors continues to be discussed, whereas the confirmation of this hypothesis requires more experimental evidence.

**Fig. 1:**
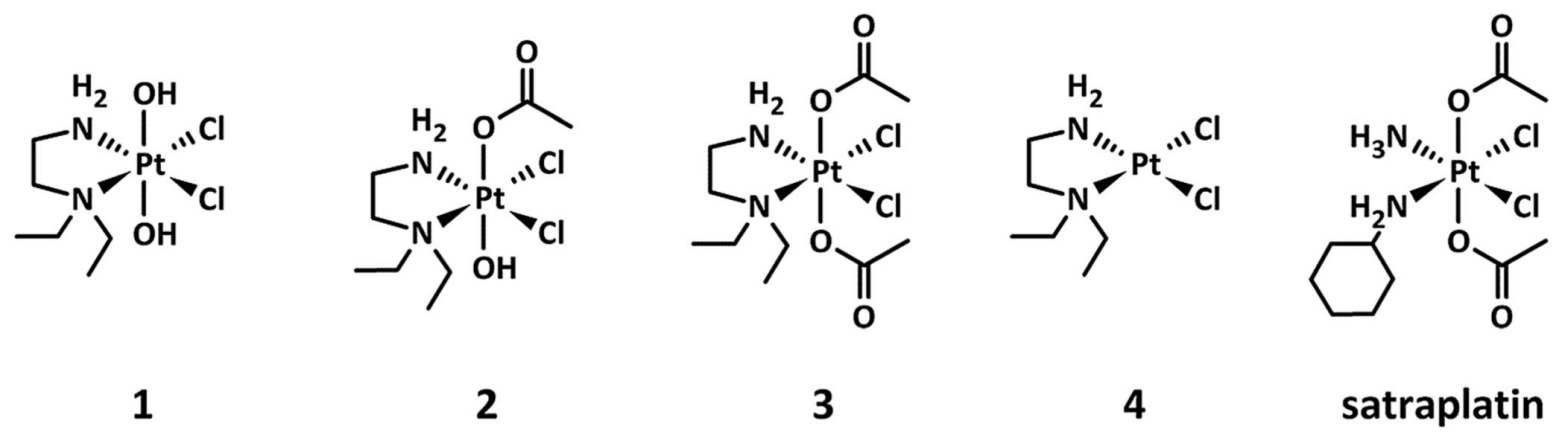
Chemical structures of the platinum(iv) complexes **1–3** under investigation, including **4** as the main platinum(ii) metabolite and the reference compound satraplatin.

This study specifically examines the influence of hypoxia on the activity of three investigational platinum(iv) compounds, their main platinum(ii) metabolite, and satraplatin as a reference compound (Fig. 1) in different *in vivo* and *in vitro* models of human cancer.

The investigated platinum(iv) complexes have the same equatorial ligand sphere, whereas their axial ligands are different, with *trans*-dihydroxido, *trans*-hydroxidoacetato, and *trans*-diacetato ligands for **1**, **2**, and **3**, respectively. The changes in the ligand sphere are associated with a different rate of reduction in the presence of ascorbic acid and glutathione, reduction potential and lipophilicity, whereas the possible platinum(ii) metabolites and the stability are comparable.^24^ This set of physiochemical properties make these compounds very suitable as model complexes for investigating the impact of the rate of reduction on the antiproliferative activity of platinum(iv) drugs under hypoxic conditions.

First, the investigational complexes **1–3** were tested in monolayer and hypoxic multicellular spheroid models to gain information about the dependence of their cytotoxic potencies on the cell culture conditions used. Subsequently, compounds **1** and **2** (performing best in hypoxic spheroids relative to monolayer cultures) were further tested in non-hypoxic spheroid models, hypoxic monolayer models and in a male SCID mouse model. Additionally, platinum distribution and cellular accumulation studies in spheroids gave further insights into the behavior of the compounds and their mode of action.

## Results and discussion

### Characterization of hypoxia within spheroids

Spheroids are *in vitro* tumor models, which share some characteristics with *in vivo* tumors, such as proliferation and nutrition gradients, chronic hypoxia and necrosis. Hypoxic regions and the necrotic core of spheroids with diameters of >400 μm (further referred to as “hypoxic spheroids” or “hypoxic 3D”) were visualized previously by using HIF1α immunofluorescence and PI staining, respectively.^25^ The obtained images displayed massive necrosis within the spheroid core and revealed that a region of mild hypoxia starts already at a depth as low as 30 μm beneath the spheroid surface. Spheroids with diameters of <200 μm, however, showed no evidence of hypoxia or necrosis^25^ and are therefore referred to as “non-hypoxic spheroids or non-hypoxic 3D”.

Characterization was now continued with regard to the hypoxic environment within live spheroids. For this purpose, oxygen distribution within >400 μm large spheroids was measured by an invasive microelectrode technique. Attempts to measure oxygen tension in live spheroids with microelectrodes published by other authors faced some technical problems and raised issues for critical discussion. For example, the steady-state measurements reported by Mueller-Klieser *et al.* took 1 min per step, with a total measuring time of 20 min per spheroid.^26^ In this time period, it is likely that passive diffusion affects the data. To bypass this problem, we used novel technical equipment, which allows the completion of the measurement within 4 s per step, with a total measurement time of about 1.5 min per spheroid.

**Fig. 2:**
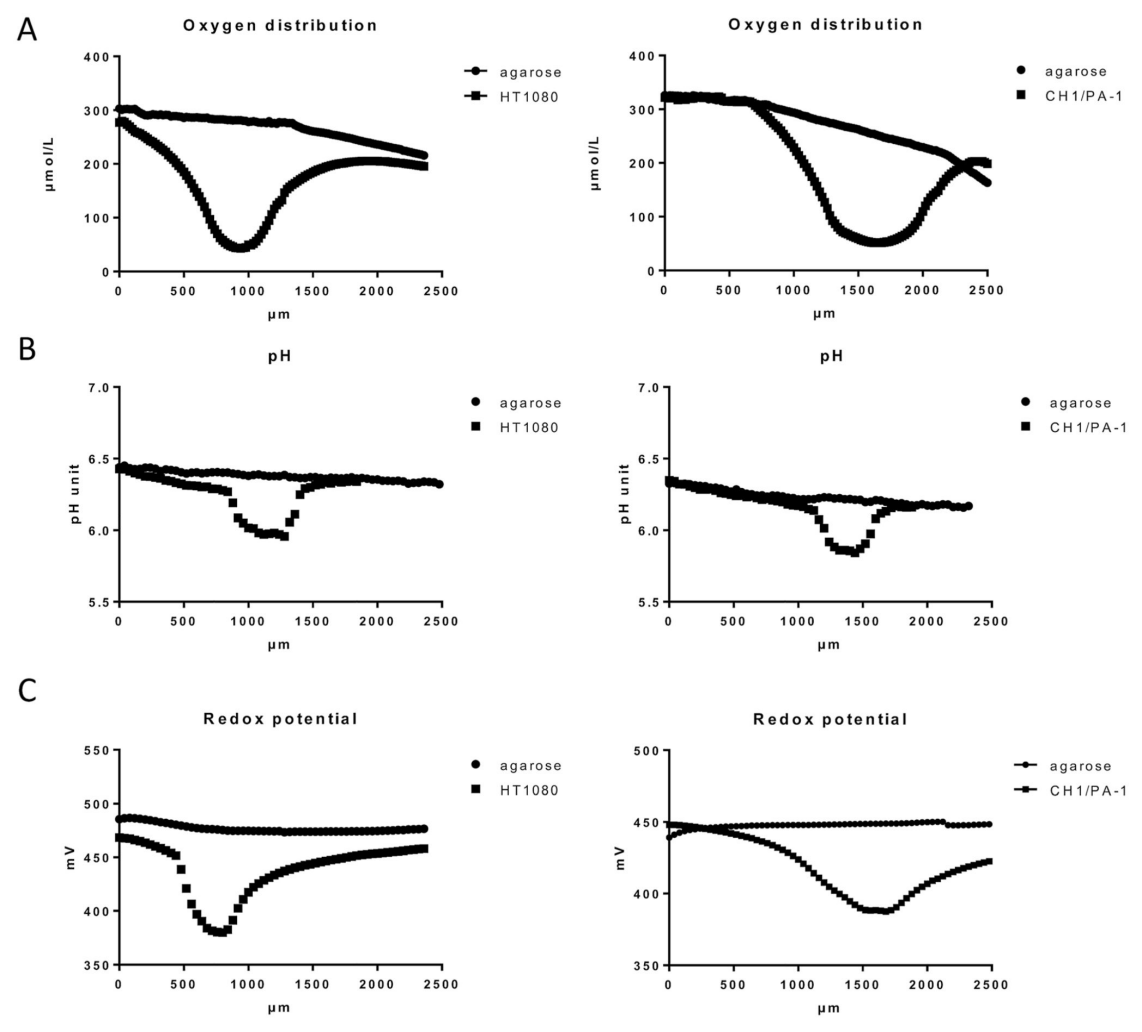
Physicochemical gradients through agarose-embedded HT1080 and CH1/PA-1 multicellular spheroids (>400 μm diameter) in comparison to agarose medium distant from the spheroid, measured by microelectrodes and exemplified by representative samples. (A) Oxygen distribution (measured in 20 μm intervals), (B) pH (40 μm intervals), (C) redox potential (40 μm intervals). Profile depths are relative to the agarose surface, whereby spheroid location relative to the agarose surface varies.

Our results featured oxygen gradient towards the necrotic core of the spheroid, with the lowest oxygen tension in the spheroid center, as shown here in HT1080 and CH1/PA-1 spheroids (Fig. 2A and ESI†). These results excellently correspond with the data published by other authors.^26^,^27^ Comparable patterns have been monitored for all investigated cell lines and samples (at least 9 samples per cell line); but small variations in absolute values depending on the cell line and therefore on the tightness of the spheroids were observed as well as individual differences between spheroids of the same cell line. Fig. 2 shows exemplary results for HT1080 and CH1/PA-1 spheroids. The experimental setup required sample immobilization and preservation of humidity and temperature; for this purpose, samples were embedded in agarose. A markedly decreasing oxygen level was noticeable already in the surrounding of the spheroid, but not in the agarose profile measurement without spheroids (used here as negative control), which only showed a very slow decrease of oxygen in deeper layers of agarose.

Hypoxia is usually accompanied by a significant drop in pH (acidosis), which is regularly observed in solid tumors^28^,^29^ and multicellular spheroids.^30^ As pH is a major factor in drug stability for most chemotherapeutics, we investigated if and to which extent a pH gradient is measurable in our samples by using the microelectrode method. The results showed a pH gradient towards the spheroid center following the oxygen distribution pattern but hardly extending to the surroundings of the spheroid (Fig. 2B and ESI†). The observed pH difference of ~0.2 pH units between the spheroid surface and the spheroid center corresponds very well with the results published previously by other authors.^30^ pH profiles give a rough idea about the dimension of the spheroids (>400 μm), whereas oxygen gradients are flatter and extend much more into the surrounding agarose, most likely due to diffusion following continued oxygen consumption by the viable spheroids.

Furthermore, hypoxia was reported to cause a significant decrease of the reduction potential in cancer cells grown in monolayers.^31^ While it is to be expected that massive hypoxia in a spheroid core will also cause a significant decrease of the reductive potential, measurements have not been reported so far. To evaluate the redox potential within the used spheroid models, microelectrode measurements in live spheroids were conducted. Our data demonstrate a considerable drop of the redox potential towards the spheroid center similar to the oxygen and pH distribution patterns (Fig. 2C and ESI†). Unfortunately, the experimental setup does not allow a distinction between intracellular and extracellular regions. Overall, the decreased oxygen level, pH and reduction potential emphasize the presence of a reductive milieu within the spheroids, most pronounced in the necrotic core (Fig. 2 and ESI†).

### Antiproliferative activity

Investigation of the antiproliferative activity in cancer cell lines is the first crucial step in exploring the anticancer potential of experimental compounds. Therefore, we studied the cytotoxicity of compounds **1–4** and satraplatin and compared them to oxaliplatin (*l*-OHP) (data published previously^25^) in different hypoxic and non-hypoxic cell culture models using cell lines with varying sensitivity for platinum(ii) drugs, including CH1/PA-1 ovarian teratocarcinoma (most sensitive), HCT116 colon cancer (moderately sensitive), and HT1080 fibrosarcoma (insensitive) cells. In the first step, the IC_50_ values were determined in hypoxic spheroids and non-hypoxic monolayer cultures by means of the Alamar Blue assay. In the non-hypoxic monolayer culture, all complexes showed the highest activity in the sensitive cell line CH1/PA-1 with IC_50_ values mostly in the low micromolar range, whereas the lowest activity was observed in the resistant cell line HT1080. In general, the activity of the complexes increases in the following order in all three cell lines: **1** < **4** < **2** < **3** < satraplatin < *l*-OHP, indicating that the reference compounds satraplatin and oxaliplatin are the most cytotoxic in monolayer culture. This order of investigational compounds **1–3** corresponds very well with the lipophilicity of these substances, with **1** being the least lipophilic and **3** the most lipophilic. These data are in very good accordance with the previously published results (compare ref. 24). The slightly divergent values in the cell line CH1/PA-1 compared to those published in ref. 38 are probably caused by the application of different reagents for staining of viable cells (MTT *vs.* Alamar Blue).

In the hypoxic spheroid (3D) model (spheroids with diameters of >400 μm, positive for HIF1α and PI staining), cytotoxicity manifested differently to extents depending on the compound. In the CH1/PA-1 spheroid model, the activity of compounds **1–4** shifted to even lower IC_50_ values, whereas satraplatin and oxaliplatin were negatively affected. These results underline the sensitivity of the cell line CH1/PA-1, as in the less sensitive cell lines HT1080 and HCT116 most IC_50_s were shifted to higher values, with the only exception of **1** in HT1080 cells. When comparing the results obtained in the 3D CH1/PA-1 model and the rate of reduction, it becomes obvious that the cytotoxic potency of the slowly reducing substance **1** (factor 6.8) benefits most, whereas the faster reducing **2** and **3** (factors 1.6 and 1.7, respectively) show only a minor activation under hypoxia. Though in the less sensitive cell lines HT1080 and HCT116 all cytotoxic potencies are negatively affected, factors decrease in a similar order: **1** (factors 1.3 and 0.6) > **2** (factors 0.7 and 0.3) > **3** (factors 0.4 and 0.1).

Surprisingly, the platinum(ii) species (**4**) showed a 1.6-fold activation in the CH1/PA-1 spheroid model, whereas the cytotoxicity of satraplatin and oxaliplatin is diminished. As this compound contains platinum already in the more reactive oxidation state +ii, the observed activation of complex **4** in the CH1/PA-1 model cannot be explained by reduction. But remarkably, the platinum(ii) reference compound oxaliplatin (*l*-OHP) did not exhibit a comparable activation. The most important structural differences between oxaliplatin and **4** are the leaving groups: one chelating oxalate group in contrast to two chlorides, respectively. These initial ligands have to be released by aquation prior to binding of the platinum(ii) center to DNA. Therefore, the rate of aquation influenced by these ligands may be one possible explanation for the activation of complex **4**. This hypothesis is supported by the previously published results by Knox *et al.* that cisplatin has a 112-fold faster hydrolysis than oxaliplatin.^32^ Furthermore, pH and hence the pH-gradient in 3D-models are crucial for the hydrolysis process and the subsequent DNA binding of platinum(ii) species, as it was reported that at pH > 8 the aqua species is transformed into the less reactive hydroxido counterpart, whereas a pH < 8 increases the binding kinetics.^33^ Nevertheless, additional investigations will be necessary to clarify the relationship of hypoxia and platinum(ii) compounds.

Since oxaliplatin, as a platinum(ii) reference compound, showed up to 10-fold increased IC_50_ values when comparing the cytotoxicity in 3D with that in 2D experiments (Table 1), we hypothesized that the observed increase in cytotoxic activity of platinum(iv) prodrugs might reflect their activation by reduction. To support this consideration, compounds **1** (lowest reduction rate and lipophilicity) and **2** (intermediate reduction rate and lipophilicity), as the best performing compounds according to the order of activation in hypoxic spheroids of all investigated cell lines, were chosen for further investigations in non-hypoxic spheroids and hypoxic monolayer culture as well as in hypoxic xenografts.

**Fig. 3:**
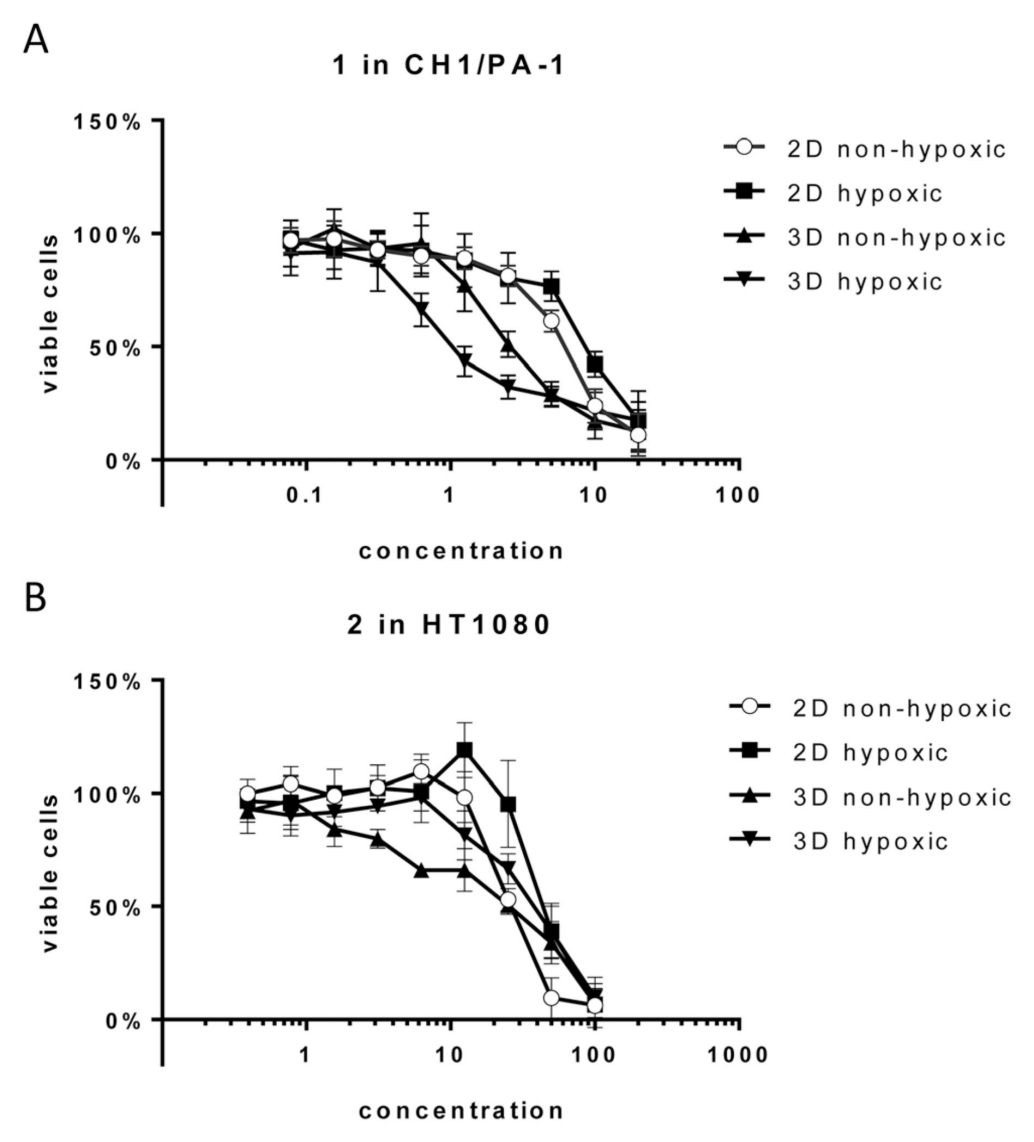
Cytotoxicity of compounds **1** and **2** in hypoxic *versus* non-hypoxic spheroids and hypoxic *versus* non-hypoxic monolayer culture. (A) Concentration-effect curves of **1** in CH1/PA-1 cells. (B) Concentration-effect curves of **2** in HT1080 cells. Values are means ± SDs from at least three independent experiments performed in triplicate.

Unexpectedly, hypoxia in monolayer culture does not elicit the activation of compounds in the sensitive cell line CH1/PA-1, while in the less sensitive cell lines HT1080 and HCT116 IC_50_ values are increased, comparable to those obtained in hypoxic spheroid culture (Fig. 3 and Table 2). These results underline the difference between hypoxia in monolayer culture and spheroids, with monolayers being appropriately supplied with nutrients, showing an early response to the lack of oxygen, but no signs of necrosis or apoptosis (results not shown) and other pH distribution and protein expression patterns.^34^,^35^

For **1** in the non-hypoxic spheroid model (spheroids with diameters of <200 μm, negative for HIF1α and PI staining) of CH1/PA-1, the IC_50_ value is intermediate between those obtained in monolayer culture and hypoxic spheroids (hypoxic 2D > non-hypoxic 2D > non-hypoxic 3D > hypoxic 3D) (Fig. 3). For **2**, IC_50_ values in hypoxic and non-hypoxic CH1/PA-1 spheroids are comparable, indicating the activation of both compounds in both spheroid models compared to monolayer culture (Table 2). The same applies for **1** and **2** in non-hypoxic HT1080 spheroids, with the IC_50_ value in the latter being comparable to that in the hypoxic spheroid model. In HCT116 cells, however, IC_50_ values in non-hypoxic spheroids are comparable to those obtained in monolayer culture. In general, these observations correspond to the results obtained in hypoxic spheroids, showing again the high sensitivity of CH1/PA-1 cells compared to HCT116 and HT1080 cells and a similar order of activation: **1** ≥ **2**.

### Drug accumulation and distribution studies

**Fig. 4:**
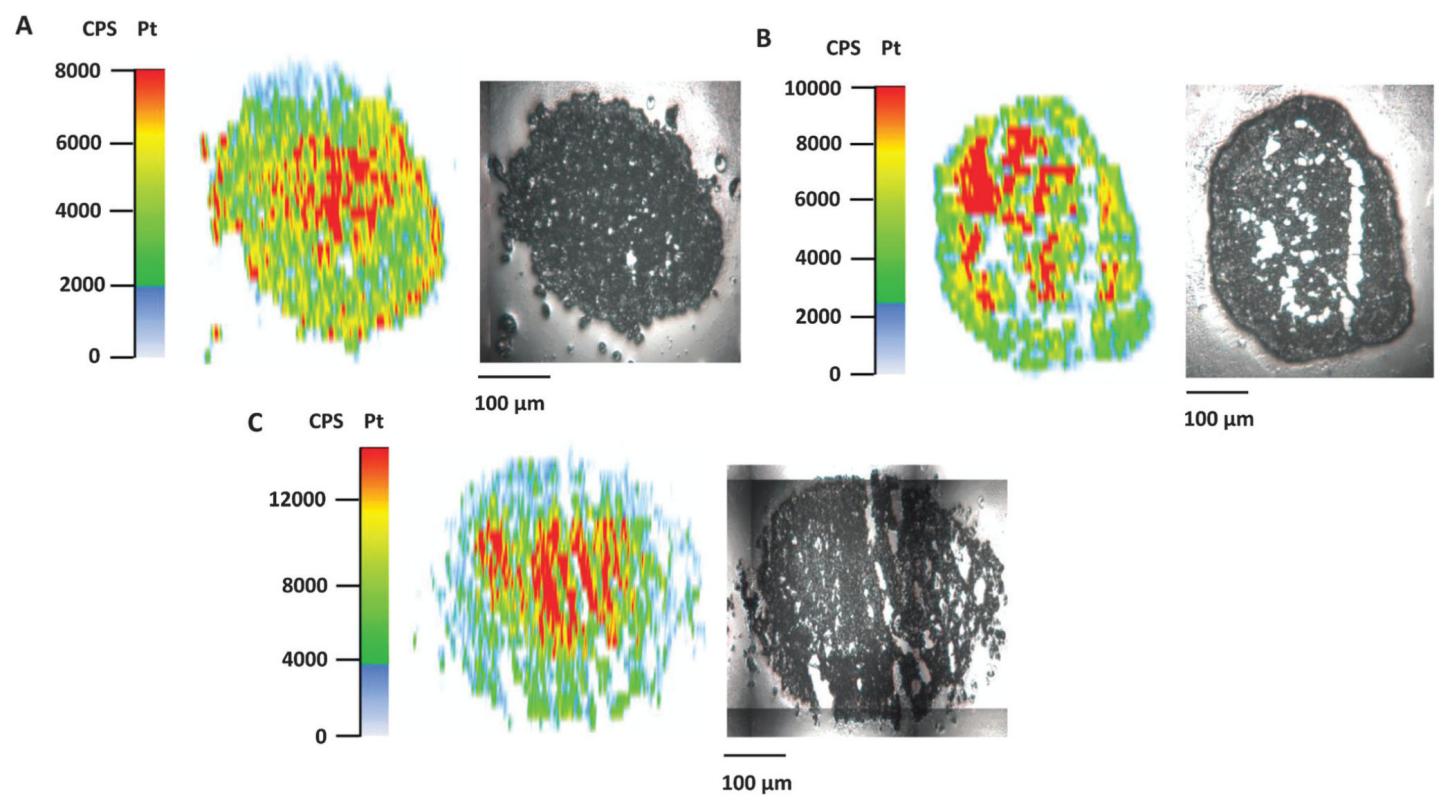
Platinum distribution in hypoxic HT1080 spheroids upon treatment with (A) compound **1**, (B) compound **2** and (C) satraplatin. Laser ablation-ICP-MS measurements were performed using 20 μm thin cryosections.

Poor drug uptake and penetration were discussed as factors for the decreased activity of different compounds in spheroid models.^36^,^37^ To shed light on the relationship between drug penetration and different cytotoxicities for the platinum complexes under investigation, platinum distribution studies were performed for **1**, **2**, and satraplatin in hypoxic HT1080 spheroids by using LA-ICP-MS. All compounds showed comparable penetration of the hypoxic HT1080 spheroids with Pt accumulation hot spots in the necrotic spheroid core (Fig. 4A–C), indicating a high affinity of the platinum complexes to hypoxic regions and underlining the possible targeting effect to these regions. Furthermore, we clearly showed that drug penetration is not a reason for differences in the cytotoxicity of platinum(iv) complexes in the HT1080 spheroid model.

Additionally, the data of total drug accumulation into hypoxic spheroids showed that **3** is taken up more effectively by all three cell lines than the other platinum compounds (Table 3). This finding correlates with the cytotoxicity data (Table 1), as compound **3** has the lowest IC_50_ values in spheroids as well as in monolayer culture. In general, the amount of platinum accumulation correlates perfectly with the lipophilicity of the compounds, as the order of lipophilicity is equivalent to the order of increased accumulation, with **3** (most lipophilic) > **2** > **1** (least lipophilic) (Table 3). However, neither lipophilicity nor accumulation corresponds to the activation in hypoxic spheroids. Although compound **1** showed 7-fold activation and compound **2** 2-fold activation in hypoxic CH1/PA-1 spheroids, compound **1** accumulates less effectively than compound **2**. In particular, **3**, with the highest accumulation, is comparably active in hypoxic CH1/PA-1 spheroids as **1**.

In general, accumulation studies in hypoxic spheroids of all studied cell lines showed the following order of accumulation: **3** > **2** > **4** > **1**. IC_50_ values, obtained in hypoxic HT1080 (**3** > **2/4** > **1**), HCT116 (**3** > **2** > **4/1**) and CH1/PA-1 (**3/1** > **2/4**) spheroids, in general correspond to the order of accumulation, in line with compound 3 as the most cytotoxic one. The order of activation in hypoxic spheroids if compared with monolayer culture, however, shows no correlation with accumulation data. The order of activation in hypoxic spheroids (HT1080 (**1** > **2/4** > **3**); HCT116 (**1** > **4** > **2** > **3**); CH1/PA-1 (**1** > **4** > **2/3**)) rather underlines that compound **3** with the highest accumulation shows the lowest activation under hypoxia, while compound **1** with the lowest accumulation is activated under hypoxia to the highest extent. These results suggest that activity under hypoxia is not highly dependent on drug accumulation and lipophilicity, but seems to be dependent on the rate of reduction of the applied platinum(iv) compounds.

### Animal studies

**Fig. 5:**
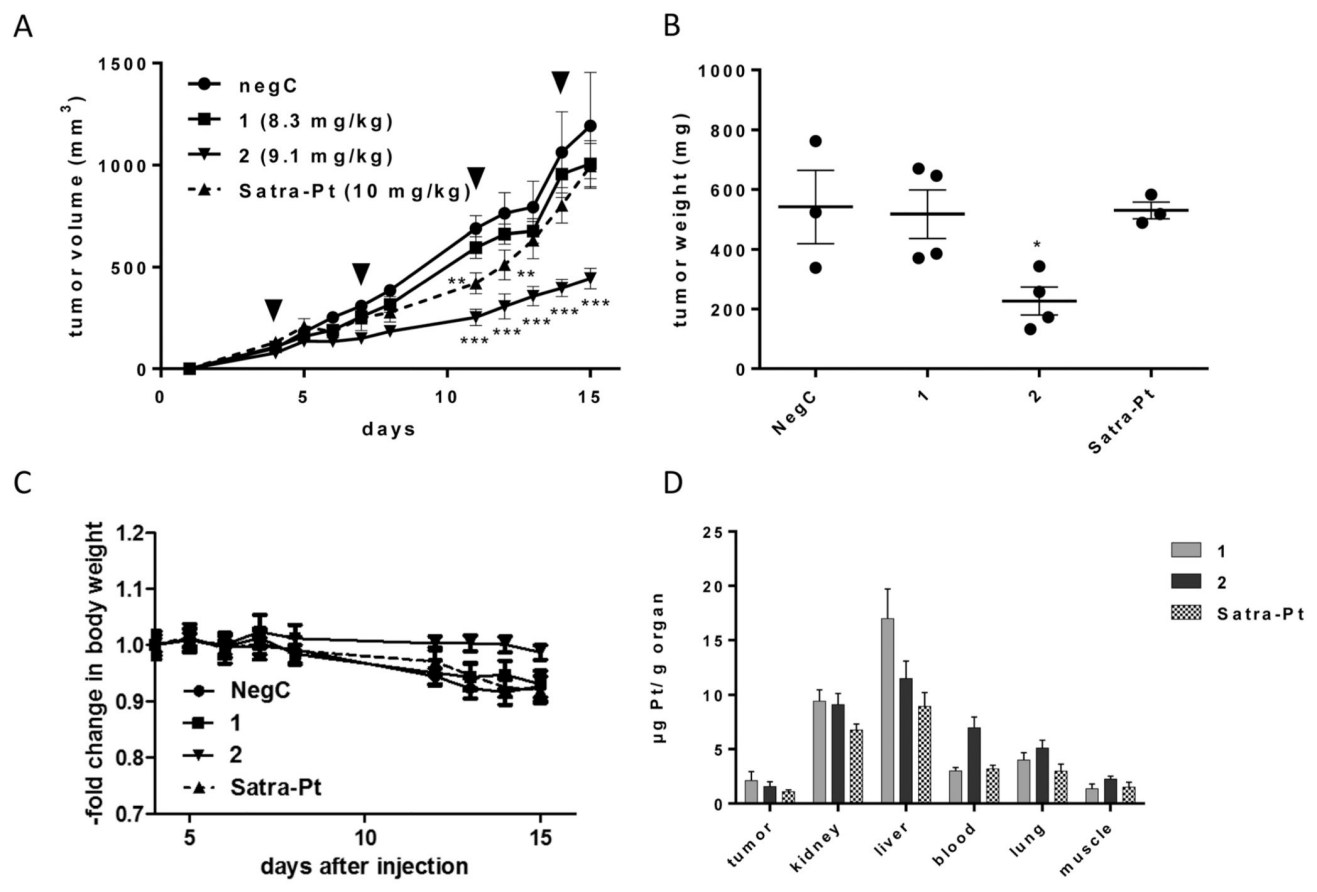
(A) *In vivo* activity of equimolar doses of **1**, **2**, and satraplatin in male SCID mice with subcutaneous tumors of human HT1080 cells (*n* = 4, treatment i.p. on days 4, 7, 11, 14 (indicated by black arrows)), ** *p* < 0.01 and ***p < 0.001 by two-way ANOVA. (B) Tumor weight compared to negative control for all treatment groups at day 15, significantly different to control: *p < 0.05 by one-way ANOVA. In the negative control and the satraplatin group, tumor weights of only 3 animals could be determined due to tumor ulceration in one animal each on day 13 and 14, respectively. (C) Body weight from all treatment groups. (D) Platinum concentration (mean ± SD) from **1**, **2** and satraplatin in mouse tissues, blood pellet and tumor, collected at day 15, determined by ICP-MS.

**1, 2**, and satraplatin (as a reference compound) were further investigated in human xenografts using SCID mice. The less sensitive HT1080 cell line was chosen as the most suitable for xenograft experiments, due to decreased or comparable IC_50_ values of both compounds in the hypoxic spheroid model and good applicability in the mouse model. Compounds were administered in equimolar doses (8.3 mg for compound **1**, 9.1 mg for compound 2 and 10 mg for satraplatin) on days 4, 7, 11, and 14. Satraplatin caused only a transient growth delay, with reduced tumor burden on days 11 and 12 (**p < 0.01 two-way ANOVA, Fig. 5A). However, this was followed by tumor regrowth resulting in nearly no difference in the tumor burden compared to control animals at day 15. With regard to our novel platinum(iv) drugs, the slowly reducing complex **1** did not display any anticancer activity. In contrast, the easiest reducible substance **2** significantly reduced tumor growth (***p < 0.001 by two-way ANOVA) from day 11 (Fig. 5A) and caused significantly reduced tumor burden (*p* < 0.05, two-way ANOVA) at day 15 (Fig. 5B). This is also reflected in body weight, as the rapid tumor growth of HT1080 cells is associated with a slow decline of body weight, which is not observed in 2-treated animals bearing reduced tumor burden (Fig. 5C).

Comparing the order of *in vivo* activity (**1** < satraplatin < **2**) with the physicochemical properties of the substances, no relation to the lipophilicity is evident, whereas the role of the rate of reduction seems consistent, with an intermediate reduction rate being highly favorable for *in vivo* activity. Furthermore, platinum levels in the tumor tissue are not the solely decisive factor, as **2** is not accumulated to the highest degree, but rather intermediate between those of **1** and satraplatin (Fig. 5C). All compounds accumulate in the kidney, suggesting renal elimination, but, remarkably, the least lipophilic compound **1** has the highest accumulation in the liver. An increased platinum concentration from **2** is found in the blood stream, suggesting a higher retention in the circulatory system compared to **1** and satraplatin.

### Platinum distribution in tumor sections analyzed by LA-ICP-MS

Bioimaging by LA-ICP-MS has been shown to be advantageous to assess the platinum distribution in histologically heterogeneous structures, compared to measurements of the average platinum concentration in tissues by ICP-MS. HT1080 tumors of SCID mice after equimolar treatment with compounds **1, 2** or satraplatin were prepared for LA-ICP-MS measurements to determine the platinum accumulation at a microscopic level. The platinum distribution was correlated with histology (visualized by an H&E stain of a consecutive slide) and with the hypoxic regions which were co-localized by pimonidazole staining in confocal microscopy. Simultaneous detection of necrotic regions was not possible due to fixation steps required for pimonidazole staining.

**Fig. 6:**
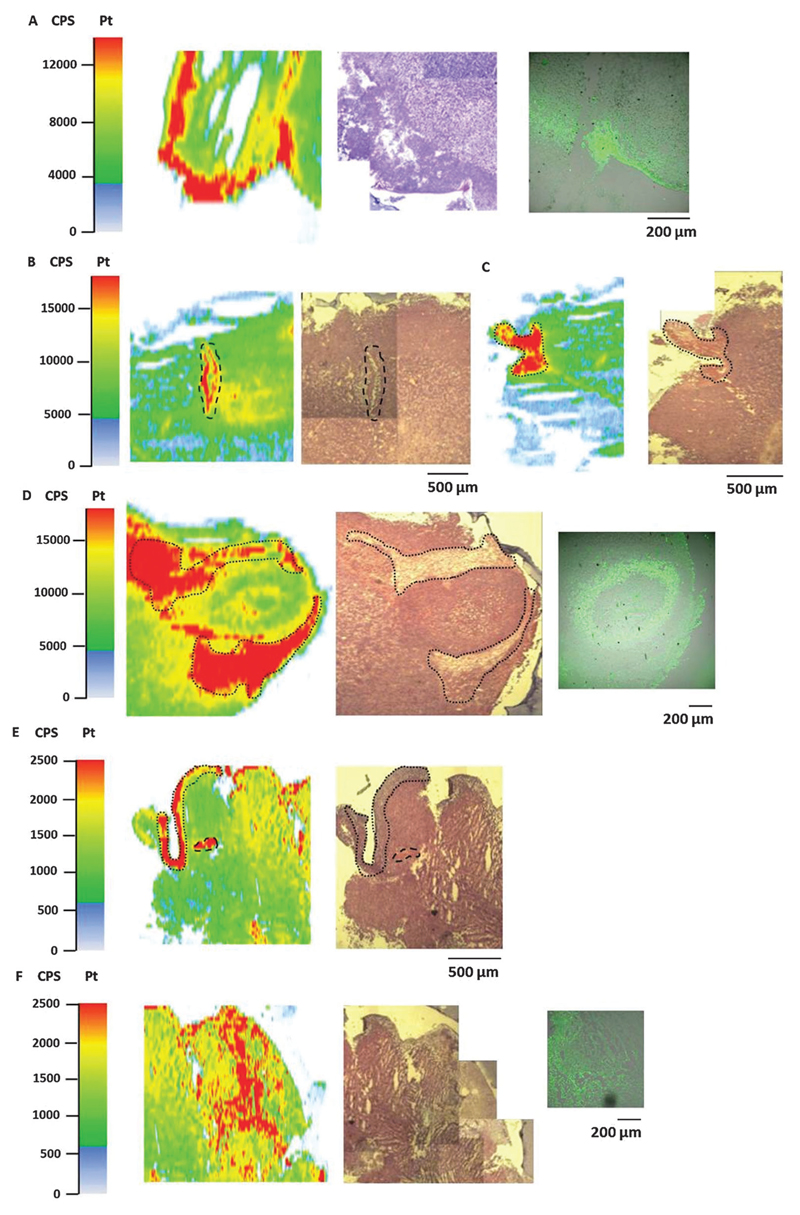
Platinum distribution in HT1080 tumor sections measured by LA-ICP-MS. Detailed views of ablated tumor samples after treatment with compounds **1** (A), compound **2** (B–D) and satraplatin (E and F); laser platinum image (left), H&E-stained section (middle) and hypoxic regions, visualized by pimonidazole staining (right). The dotted spots in the laser image and H&E-stained slides correspond to loose, connective tissue with sparsely scattered tumor cells. The dashed lines indicate the presence of a blood (micro-)vessel.

In general, a heterogeneous platinum distribution was observed in the ablated tumor sections after treatment with the respective platinum compounds. The areas of platinum enrichment (indicated by red color) most likely correspond to the localization of blood (micro-)vessels (Fig. 6B and E) and to loose, soft tissue (which is only sparsely infiltrated with tumor cells) as well as to striated muscle of the treated mouse (Fig. 6C–E). Malignant tissue, which is composed of denser packed tumor cells, in contrast, generally exhibited lower platinum levels. These results are in accordance with previous LA-ICP-MS studies on platinum complexes in tumors of CT26-colon cancer bearing mice.^39^

In detail, for the tumor sections after treatment with compound **1** (Fig. 6A) and satraplatin (Fig. 6F) a correlation with platinum enrichment and hypoxic regions was observed. The ablated tumor sample after treatment with compound **2** revealed the presence of a tumor nodule (confirmed by the underlying histology). Again, areas of loose, soft tissue surrounding the tumor nodule comprised increased platinum amounts (Fig. 6D). Still, platinum was also highly concentrated in the tumor nodule, where the histology indicates the presence of a central necrotic region. Interestingly, pimonidazole co-localized here with a rim of platinum enrichment around the central necrosis. These results are in agreement with the laser ablation data of the HT1080 spheroids with increased platinum levels in the core and reveal the ability of platinum species to penetrate into these regions *in vitro* as well as *in vivo*.

## Conclusion

Hypoxia-induced activation of platinum(iv) drugs in solid tumors is an attractive strategy for targeting anticancer therapies. Therefore, investigations were performed on the relationship between physicochemical properties of three platinum(iv) complexes, their platinum(ii) counterpart and satraplatin as a reference complex and the cell biological responses in non-hypoxic and hypoxic 2D as well as 3D tumor cell culture models. Subsequently, the findings were underpinned by *in vivo* experiments in a SCID male mouse model.

Although a strong correlation could be found between lipophilicity, drug accumulation and 2D and 3D cytotoxicity, the extent of drug accumulation is hardly related to the activation under hypoxia in hypoxic spheroids and the SCID mouse model. Rather, activity seems to be dependent on the rate of reduction of these complexes, although data suggest an activation even for the platinum(ii) species **4** in one of the three spheroid models. Complex **1** with the lowest rate of reduction is least active in all tested cell lines and settings, whereas the activities of the fastest reducing compound **3** seem to be affected by too fast biotransformation in hypoxic 3D models, especially in less sensitive cell lines. On the other hand, compound **2**, with an intermediate lipophilicity and reduction rate, seems to have optimal properties for high efficacy. This complex elicited a significant inhibition of tumor growth *in vivo* superior to **1** and satraplatin. To the best of our knowledge, we are the first to show the activation of platinum(iv) complexes in a 3D CH1/PA-1 *in vitro* model compared to the corresponding 2D model. These findings indicate an advantage of these platinum(iv) compounds over satraplatin.

## Material and methods

### Cell lines and culture conditions

The human cell line CH1 (identified *via* STR profiling as PA-1 ovarian teratocarcinoma cells by Multiplexion; see also ref. 40) was kindly provided by Lloyd R. Kelland (CRC Centre for Cancer Therapeutics, Institute of Cancer Research, Sutton, UK), and the colon cancer cell line HCT116 (ATCC^®^ CCL-247™) was purchased from American Type Culture Collection (ATCC). These cell lines were grown in MEM (supplemented with 10% heat-inactivated fetal bovine serum (Life Technologies, Austria, Vienna), 1% l-glutamine, 1% sodium pyruvate, 1% non-essential amino acids solution (all from Sigma Aldrich, Austria, Vienna)). Human HT1080 (ATCC^®^ CCL-121™) fibrosarcoma cells and CCD-18Co normal colon fibroblasts were obtained from ATCC (CRL1459) and maintained in MEM (Sigma) supplemented with 10% FCS. Monolayer cultures of all cell lines were grown in cell culture treated 75 cm^2^ flasks (Starlab, Hamburg, Germany).

### Spheroid culture

For spheroid production, CH1/PA-1, HCT116, and HT1080 cells were harvested from culture flasks by trypsinization and seeded in MEM using non-cell culture treated round bottom 96-well plates (VWR) in densities of 1 × 10^4^ (CH1/PA-1), 2 × 10^3^ (HCT116), and 8 × 10^3^ (HT1080) viable cells per well, respectively, for spheroids with >400 μm diameter 7 days prior to treatment; and in densities of 1 × 10^3^ (CH1/PA-1), 0.3 × 10^3^ (HCT116), 0.3 × 10^3^ (HT1080) viable cells per well, respectively, for spheroids with <200 μm diameter 3 days prior to treatment. To minimize evaporation, the outermost wells were not used for spheroid production and filled with 200 μL of PBS instead. Cultures were maintained at 37 °C in a humidified atmosphere containing 95% air and 5% CO_2_.

### Compounds

Compounds (**1, 2, 3, 4,** satraplatin and oxaliplatin (*l*-OHP)) have been synthesized at the Institute of Inorganic Chemistry, University of Vienna, according to literature procedures.^38^ All platinum(iv) compounds were dissolved in MEM to stock solutions of 2 mM and then diluted in MEM to the required concentrations. Cisplatin and **4** were dissolved shortly before usage in MEM to a stock concentration of 0.4 mM and diluted further in MEM.

### Alamar Blue assay

For tests with monolayers, CH1/PA-1, HCT116, and HT1080 cells were harvested from culture flasks by trypsinization and seeded in MEM into 96-well microculture plates (Falcon) 24 h prior to treatment in densities of 1 × 10^3^ (CH1/PA-1), 2 × 10^3^ (HCT116), 3 × 10^3^ (HT1080) viable cells per well, respectively. For tests in 3D cultures, spheroids with the required diameter were treated directly in culture plates.

For both monolayer and spheroid treatment, stock solutions of the test compounds were prepared and diluted stepwise to obtain a dilution series. 100 μL of dilution were added to each well, and plates were incubated for 96 h at 37 °C and 5% CO_2_. A fresh solution of 440 μm (≈110 μg mL^–1^) resazurin sodium salt (Sigma Aldrich) in PBS was prepared and 20 μL of it were added to each well. Plates were stained at 37 °C and 5% CO_2_ for 4 h (monolayer experiments) or overnight (spheroid experiments).

### ICP-MS

#### Chemicals

Milli-Q water (18.2 MΩ cm, Milli-Q Advantage, Darmstadt, Germany) was used for all dilutions for ICP-MS measurements. Nitric acid (≥65%, p.a., Fluka, Buchs, Switzerland) was further purified in a quartz sub-boiling point distillation unit (Milestone-MLS GmbH, Leutkirch, Germany). Platinum and rhenium standards for ICP-MS measurements were purchased from CPI International (Amsterdam, The Netherlands). Tissue-Tek medium (Sakura Finetek, The Netherlands) was used for embedding the cryosections. All other reagents and solvents were obtained from commercial sources and were used without further purification.

#### ICP-MS measurements

The total platinum content in spheroids and in mouse tissues was determined by using an ICP-quadrupole MS instrument Agilent 7500ce (Agilent Technologies, Waldbronn, Germany). The ICP-MS instrument was equipped with a CETAC ASX-520 autosampler (Nebraska, USA) and a MicroMist nebulizer at a sample uptake rate of approx. 0.25 mL min^−1^. The instrument was tuned daily and rhenium served as internal standard for platinum to account for instrumental fluctuations and matrix effects. The instrumental parameters are summarized in Table 4. The Agilent MassHunter software package (Workstation Software, Version B.01.01, 2012) was used for data processing.

In the case of bioimaging by LA-ICP-MS, data were recorded by using a Triple Quadrupole ICP-MS Agilent 8800 (Agilent Technologies, Tokyo, Japan) using the instrumental parameters given in Table 4.

#### Sample preparation for accumulation studies (ICP-MS)

Spheroids were treated with 20 μM of compounds **1–4** for 4 h, then harvested and transferred into 1.5 mL Eppendorf tubes (10 spheroids with an average diameter of 450 μm per tube). After 3× washing with 1 mL PBS, spheroids were lysed with 400 μL HNO_3_ for 1 h and filled up with Milli-Q water to a final volume of 8 mL. The total platinum content was determined with ICP-MS using the instrumental parameters summarized in Table 4.

#### Determination of the platinum concentration in mouse tissues by ICP-MS

Digestion of mouse tissue samples (tumor, kidney, liver, lung, muscle) as well as blood pellets was performed with sub-boiled nitric acid by using a microwave system Discover SP-D (CEM Microwave Technology, Germany). The following microwave parameters were used: temperature: 200 °C; ramp time: 4 min; hold time: 6 min; maximal power: 300 W. Digested samples were diluted with Milli-Q water resulting in nitric acid concentrations lower than 3% and platinum concentrations lower than 15 ng g^−1^. The total platinum content was determined with ICP-MS using the instrumental parameters given in Table 4.

#### Bioimaging in multicellular spheroids and mouse tumor samples by LA-ICP-MS

After compound treatment with 1–5 μM (sub-cytotoxic concentrations) for 96 h, spheroids were harvested and transferred into 1.5 mL Eppendorf tubes (10–20 spheroids per tube) and washed 3× with PBS. After the last washing step, PBS was removed completely and Eppendorf tubes were carefully filled up with 0.5–1 mL Tissue Tek (Sakura) prior to freezing at −80 °C. Spheroids were marked with 1 μL of trypan blue. Subcutaneous xenografts were removed and frozen at −80 °C. For LA-ICP-MS measurements, samples were cryosectioned into slices of 20 μm thickness by using a cryotom (Microm HM 550, Thermo Fischer), placed onto glass slides and air dried. A consecutive slice of 5 μm of the respective organ was stained with hematoxylin–eosin (H&E) and used for histological evaluation.

A Nd:YAG solid state laser (NWR 213, ESI, Fremont, CA, USA) at a wavelength of 213 nm was used to obtain the spatially resolved distribution of platinum in spheroids and tumor sections. An optical sample map of the region of interest was generated prior to the measurement. The output laser energy was tuned separately for every sample in order to ensure complete ablation of the sample material. Ablation was performed using parallel line scans and a spacing of 10 μm between the lines. For tumor sections, a frequency of 10 Hz, a spot size of 70 μm and a scan speed of 40 μm s^−1^ was used, whereas for spheroids the laser parameters were adapted to 20 Hz, a spot size of 10 μm and a scan speed of 10 μm s^−1^. The ablated sample material was transferred to the ICP-MS instrument with helium (quality 5.0) at a flow rate of 400 mL min^−1^. Data were recorded by using an ICP-MS Agilent 8800 and the instrumental parameters summarized in Table 4. The software Igor Pro (Wavemetrics, Igor Pro 6.34A) together with its add-on Iolite (Iolite Version 2.5) was used for further data processing and generation of platinum distribution maps. The data reduction scheme ‘Trace_Elements’ including blank subtraction and no smoothing of the visualization was applied according to the manual by the authors (Iolite User Manual, Version 2.0).^41^ The aspect ratio of the image was set according to the dimensions of the ablated area in order to obtain accurately shaped pictures.

### Hypoxia detection

Tumor slices were fixed with acetone and stained according to the Hypoxyprobe™-1 Kit (hpi) protocol. Alexa Flour 647-labeled secondary antibody (Cell Signaling, Frankfurt am Main, Germany) diluted 1:1000 in PBST was used for fluorescence detection in the confocal microscope Leica CLSM (Leica Microsystems, Wetzlar, Germany). As the spheroid sections did not stand fixation due to poor cellular cohesion and wash steps required for Hypoxyprobe™-1 Kit application, whole spheroids were stained with HIF-1 alpha antibody as described previously.^25^

### Hypoxic chamber

For hypoxia induction in monolayer culture, cells were seeded into 96-well plates and allowed to attach for 24 h prior to treatment in a hypoxic chamber (C-chamber and ProOx C21, BioSpherix) with the oxygen level lowered to 0.5% by displacement with nitrogen. Treatment of cells was performed for 96 h in the hypoxic chamber with 0.5% oxygen.

### Animal experiments

Six- to 8-week-old male CB-17 scid/scid (SCID) mice were purchased from Harlan Laboratories (San Pietro al Natisone, Italy). The animals were kept in a pathogen-free environment and every procedure was done in a laminar airflow cabinet. The experiments were done according to the regulations of the Ethics Committee for the Care and Use of Laboratory Animals at the Medical University Vienna (proposal number BMWF-66.009/0084-II/3b/2013), The U.S. Public Health Service Policy on Human Care and Use of Laboratory Animals as well as the United Kingdom Coordinating Committee on Cancer Prevention Research’s Guidelines for the Welfare of Animals in Experimental Neoplasia.

For the local tumor growth experiments, HT1080 cells (1 × 10^6^) were injected subcutaneously into the right flank. Animals were randomly assigned to treatment groups and therapy was started when tumor nodules were ~100 mm^3^. Animals were treated with **1** (i.p. 8.3 mg kg^−1^ dissolved in 5% DMSO), **2** (i.p. 9.1 mg kg^−1^ dissolved in 5% DMSO), or satraplatin (i.p. 10 mg kg^−1^ dissolved in 5% DMSO) on days 4, 7, 11, and 14. Animals in the control group received water intraperitoneally. Animals were controlled for distress development every day and tumor size was assessed regularly by caliper measurement. Tumor volume was calculated by using the formula: (length × width^2^)/2.

At day 15, animals received pimonidazole (60 mg kg^−1^ i.p. dissolved in 0.9% NaCl) and were sacrificed 30–60 min later. For this purpose, the mice were anesthetized and blood was collected by heart punctuation.

### Oxygen and redox microsensor measurements

For microsensor measurements, spheroids were washed once in sterile filtered PBS followed by resuspension in 1% agarose prepared by using 1 × PBS. The suspended spheroids were aliquoted onto a pre-heated (37 °C) Petri dish containing a 1 cm layer of PBS–agarose (1%). Samples were maintained at 37 °C during microsensor measurements in a circulating waterbath (ThermoScientific SC 100-S19T, Karlsruhe, Germany).

Oxygen and redox potential profiles were measured by using microsensors (10 μm tip diameter; Unisense, Aarhus, Denmark). Microsensor calibration and measurements were implemented according to the manufacturer instructions *via* the software interface (SensorTrace Suite, Unisense Aarhus, Denmark) provided with the microsensors. Microsensors were calibrated by using external standards from two-point standard curves generated prior to measurements.

For oxygen measurements, a saturated atmospheric oxygen reading was obtained from a vigorously aerated volume of water at 20 °C. After 5 min of aeration, the bubbling was stopped and replicate measures were recorded. The saturated oxygen concentration was calculated by using the microsensor software based upon the solution temperature and salinity. An anoxic solution was prepared containing 0.1 M sodium ascorbate in 0.1 M NaOH and replicate measures were performed. Oxygen distribution was measured with the step size of 20 μm and total measuring time per step was 4 s.

The redox microsensor was calibrated with saturated solutions of quinhydrone (10 g L^−1^) in pH standards of 4.0 and 7.0. Redox measurements were performed by using an Ag/AgCl reference electrode (REF 321 Radiometer Analytical, Denmark). Depth profiles were collected by fixing the microsensors in a 2D micro profiling system (Unisense, Aarhus, Denmark) and controlled by using the SensorTrace Suite software. Redox measurements were performed with a step size of 30 μm and total measuring time per step was 14 s.

### Statistics

All animal data are expressed as mean ± SEM. Results were analyzed and illustrated by using GraphPad Prism (version 5; GraphPad Software, San Diego, CA). Statistical analyses were performed by using one- and two-way ANOVA with drug treatment and time as independent variables, and conducted with Bonferroni post-tests to examine the differences between the different drug treatment regimens and the diverse responses. The statistical significance is either described in the respective figure legends, or indicated with asterisks (*p < 0.05; **p < 0.01; ***p < 0.001).

## Supplementary Material

† Electronic supplementary information (ESI) available. See DOI: 10.1039/c5mt00312a

si

## Figures and Tables

**Table 1:** Cytotoxicity of complexes **1–4**, satraplatin and oxaliplatin (*l*-OHP) in hypoxic spheroid (3D) models and non-hypoxic monolayer (2D) models, determined by means of the Alamar Blue assay (exposure time 96 h). Values are means ± SDs from at least three independent experiments performed in triplicate

	IC_50_ [μm]

	CH1/PA-1 2D	CH1/PA-1 3D	2D/3D	HT1080 2D	HT1080 3D	2D/3D	HCT116 2D	HCT116 3D	2D/3D
1	6.2 ± 0.6	0.90 ± 0.13	**6.8**	96 ± 2	77 ± 11	**1.3**	42 ± 9	65 ± 10	0.6
2	3.1 ± 0.6	1.9 ± 0.2	**1.6**	26 ± 2	40 ± 8	0.7	14 ± 2	51 ± 12	0.3
3	1.6 ± 0.1	1.0 ± 0.2	**1.7**	9.8 ± 2.1	21 ± 5	0.4	4.0 ± 0.4	29 ± 7	0.1
4	4.6 ± 0.9	1.9 ± 0.1	**2.4**	35 ± 8	47 ± 5	0.7	35 ± 4	71 ± 6	0.5
Satra-Pt	0.24 ± 0.04	0.40 ± 0.01	0.5	5.1 ± 0.3	7.8 ± 1.1	0.6	2.7 ± 0.6	6.0 ± 1.3	0.4
*I*-OHP^a^	0.20 ± 0.01	1.4 ± 0.2	0.2	1.7 ± 0.1	18 ± 3	0.09	0.57 ± 0.10	1.6 ± 0.2	0.4

a Taken from ref. 25.

**Table 2:** Cytotoxicity of compounds **1** and **2** in hypoxic *versus* non-hypoxic spheroids as well as hypoxic and non-hypoxic monolayer culture

	IC_50_ [μM]								
	Compound 1					Compound 2			
Cell line	2D	2D hypox.	3D	3D hypox.		2D	2D hypox.	3D	3D hypox.
CH1/PA-1	6.2 ± 0.6	8.6 ± 1.0	2.6 ± 0.4	0.9 ± 0.1		3.1 ± 0.6	2.2 ± 0.9	1.5 ± 0.2	1.9 ± 0.2
HCT116	42 ± 9	74 ± 5	27 ± 3	66 ± 10		14 ± 2	38 ± 1	17 ± 2	52 ± 12
HT1080	96 ± 22	87 ± 9	43 ± 11	77 ± 11		26 ± 2	45 ± 8	27 ± 5	40 ± 8

**Table 3:** Platinum accumulation in hypoxic spheroid models treated with **1**–**4** in relation to rate of reduction and lipophilicity of the compound. Values are means ± SDs from at least three independent experiments performed in triplicates

	Platinum accumulation in ng per spheroid				
	CH1/PA-1	HT1080	HCT116	Reduction half time^a^	Lipophilicity log k_w_ b
1	0.31 ± 0.07	0.13 ± 0.03	0.16 ± 0.03	~15 h	0.51
2	0.26 ± 0.06	0.45 ± 0.06	0.47 ± 0.08	~5 h	1.05
3	1.3 ± 0.3	1.9 ± 0.1	1.9 ± 0.3	~15 min	1.60
4	0.45 ± 0.12	0.34 ± 0.06	0.40 ± 0.04	—	—

a Obtained from ref. 24.

b Obtained from ref. 38.

**Table 4:** Instrumental parameters for (LA-)ICP-MS measurements

	ICP-MS	LA-ICP-MS
RF power [W]	1500	1350
Cone material	Nickel	Nickel
Carrier gas [l min^−1^]	0.90	1.10
Make up gas [l min^−1^]	0.20	—
Plasma gas [l min^−1^]	15	15
Monitored isotopes	^185^Re, ^194^Pt,^195^Pt	^195^Pt
Dwell time [s]	0.3	0.1
Number of replicates	10	1
