# Supplementary material for: Behavior of platinum(iv) complexes in models of tumor hypoxia: cytotoxicity, compound distribution and accumulation†
Source: Metallomics. Author manuscript; Available in PMC 2018 Sep 10. (PMC6130773; doi:10.1039/c5mt00312a)
Supplement: si [file NIHMS79172-supplement-si.pdf]

Electronic Supplementary Material (ESI) for Metallomics.
This journal is © The Royal Society of Chemistry 2016

Supplementary Information

Behavior of platinum(IV) complexes in models of tumor hypoxia: cytotoxicity, compound distribution and accumulation

Ekaterina Schreiber-Brynzak,[a] Verena Pichler,[a] Petra Heffeter,[b,c] Buck Hanson,[d,g] Sarah Theiner,[a,c] Irene Lichtscheidl-Schultz,[e] Christoph Kornauth,[f] Luca Bamonti,[a] Vineet Dhery,[b,c] Diana Groza,[b,c] David Berry,[d,g] Walter Berger,[b,c] Markus Galanski,[a] Michael Jakupec,[a,c,g*] Bernhard K. Keppler[a,c,g]

[a] University of Vienna, Institute of Inorganic Chemistry, Waehringer Strasse 42, 1090 Vienna, Austria

[b] Medical University of Vienna, Department of Medicine I, Institute of Cancer Research and Comprehensive Cancer Center, Borschkegasse 8a, 1090 Vienna, Austria

[c] University of Vienna, Research Platform "Translational Cancer Therapy Research", Waehringer Strasse 42, 1090 Vienna, Austria

[d] University of Vienna, Department of Microbiology and Ecosystem Science, Division of Microbial Ecology, Althanstrasse 14 (UZA I), 1090 Vienna, Austria

[e] University of Vienna, Core Facility Cell Imaging and Ultrastructure Research, Althanstrasse 14 (UZA I), 1090 Vienna, Austria

[f] Medical University of Vienna, Clinical Institute of Pathology, Vienna General Hospital, Spitalgasse 23, 1090 Vienna, Austria

[g] University of Vienna, Research Network Chemistry Meets Microbiology, Althanstrasse 14 (UZA I), 1090 Vienna, Austria

*Corresponding author. Tel. +43 1 4277 52610. E-mail address: michael.jakupec@univie.ac.at (M. A. Jakupec).

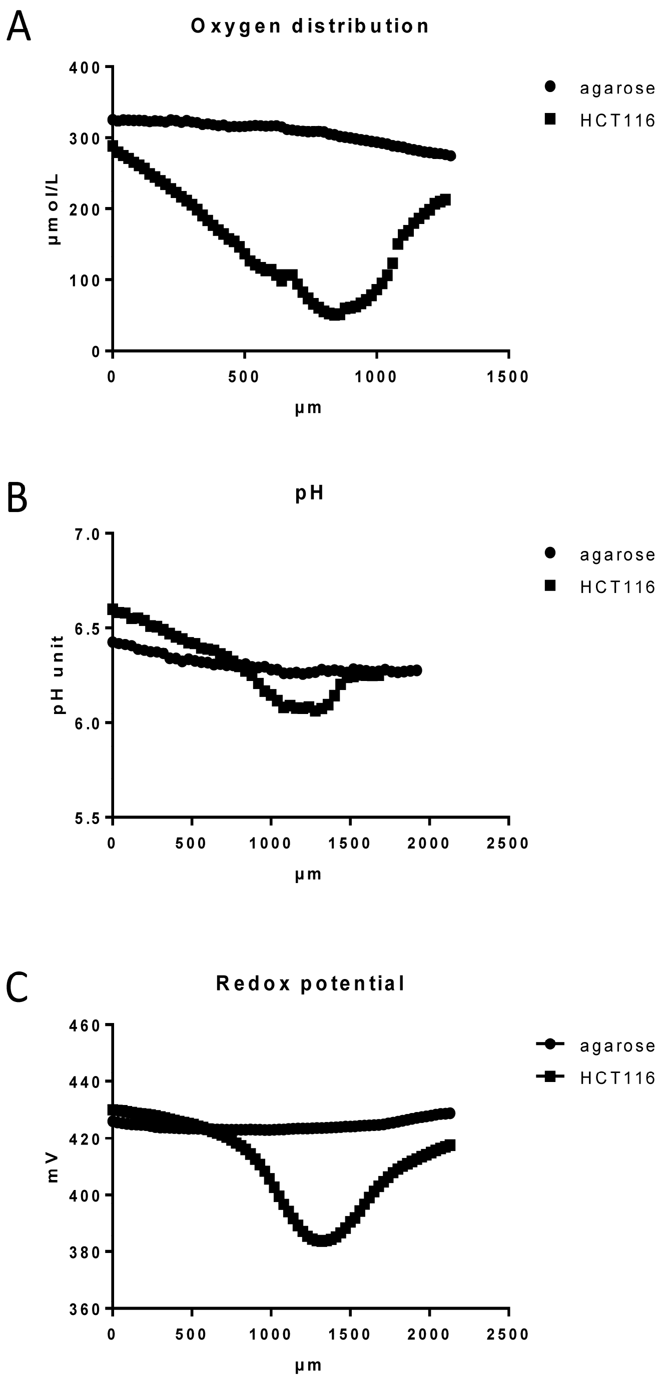

Fig. S1 A. Oxygen measurement in hypoxic HCT116 spheroids and agarose as negative control. Measurements were conducted every 20 µm. **B**. pH measurement in hypoxic HCT116 spheroids and agarose as negative control. Measurements were conducted every 40 µm. **C**. Redox potential

measurement in hypoxic HCT116 spheroids and agarose as negative control. Measurements were conducted every 40 µm.
